# Polymerization-Dependent Thermal Stability of Chinese Quince Proanthocyanidins and Their Inhibition of Heterocyclic Amines in a Simulated Frying System

**DOI:** 10.3390/foods15193404

**Published:** 2026-09-24

**Authors:** Can Fang, Mei-Qi Yang, Wan-Qing Kong, Qing Shi, Hao Liu, Lin Xiao, Xing-Yu Liu, Jia-Hao Wang, Zhao Qin, Hua-Min Liu

**Affiliations:** 1 Food Laboratory of Zhongyuan, Luohe 462300, China; fcan666@163.com; 2 College of Food Science and Engineering & Institute of Special Oilseed Processing and Technology, Henan University of Technology, Zhengzhou 450001, China; yangmq2023@163.com (M.-Q.Y.); kwq8342@163.com (W.-Q.K.); 13333822624@163.com (Q.S.); liuhao459150241@163.com (H.L.); 16650913198@163.com (L.X.); 15237193198@163.com (X.-Y.L.); 3 School of International Education, Henan University of Technology, Zhengzhou 450001, China; 231170100209@stu.haut.edu.cn

**Keywords:** Norharman, Harman, degree of polymerization, thermal stability, antioxidant activity, inhibition

## Abstract

Heterocyclic amines (HCAs), a class of potent mutagenic and carcinogenic compounds generated during the high-temperature processing of protein-rich foods, pose significant health risks to humans. Proanthocyanidins (PAs) are used to inhibit HCAs due to their low toxicity and strong inhibitory activity. However, PA activity may vary at different temperatures. In this study, PAs with different degrees of polymerization were heated at 180 °C to evaluate their thermal stability and inhibition of HCAs. With increasing temperature, the antioxidant activities of highly polymerized PAs (PPA) and oligomeric PAs (OPA) decreased. OPA demonstrated higher residual rates and antioxidant activities compared to PPA. Following simulated frying, the thermal stability of OPA and PPA decreased, while total oligomeric PA content increased. OPA exhibited stronger inhibition of HCAs than PPA, while PPA-180 (PPA after heating at 180 °C) and OPA-180 (OPA after heating at 180 °C) had a stronger inhibitory effect compared to PPA and OPA, respectively. In the HCA model system, OPA-180 stood out by exhibiting the highest inhibition rates. The study provides a foundation for the inhibition of HCAs by PAs.

## 1. Introduction

Many traditional fried meat products are very popular with consumers. However, prolonged frying can degrade food quality and produce different levels of heterocyclic amines (HCAs). HCAs are commonly generated in high-protein foods processed at high temperatures [1,2]. HCA types and reaction mechanisms can vary significantly among different foods. In foods rich in plant protein, two non-polar HCAs are produced by thermal processing reactions between tryptophan and glucose [3,4]. In foods rich in animal protein, thermal processing of tryptophan, glucose, and creatine produces non-polar HCAs (Norharman and Harman), while thermal processing of phenylalanine, glucose, and creatine yields polar HCAs (PhIP) [5,6,7]. HCAs are classified as carcinogenic or mutagenic substances based on their ability to form DNA adducts [1]. Epidemiological evidence points to a correlation between HCAs and developing prostate, breast, and colon cancers [8,9]. Therefore, regulating HCA content in fried foods is important to safeguard consumer health. Research has demonstrated that HCA formation can be suppressed by adding antioxidants, including epicatechin and various plant extracts such as pomegranate, grape seed, garlic, hawthorn, and green tea [10,11,12].

Chinese quince (*Chaenomeles sinensis*), a part of the Rosaceae family, is widely cultivated in China, Korea, and Japan [13]. Its fruit exhibits an inherently sour, astringent flavor. Currently, ~10% of harvested Chinese quince fruits undergo industrial processing, mainly as fruit wine, jam, juice, and herbal raw materials, which severely restricts its deep-value utilization. Chinese quince fruit is rich in bioactive compounds, among which polyphenols predominate. Importantly, proanthocyanidins (PAs) constitute 99.5% of these polyphenols [14]. Due to their prominent antioxidant capacity, PAs extracted from Chinese quince are a promising candidate for the mitigation of HCA formation in food systems. The strong antioxidant properties of Chinese quince PAs make them a key substance for controlling HCAs in food.

Our previous studies demonstrated that HCAs are inhibited by Chinese quince PAs in fried food [15]. Wang et al. [16] indicated that different polymerization degrees of PAs have various inhibitory effects on HCAs. However, Chinese quince fruit PAs exhibited structural diversity. Most Chinese quince PAs are highly polymerized, with only a few oligomers and monomers [14]. Many factors (e.g., pH, temperature, metal ions, type of fat, etc.) in the food system could cause chemical changes in PAs, leading to instability [17]. During the high-temperature process of frying, PAs can be easily degraded in food due to their instability, causing structural changes and rapidly transforming into a variety of derivatives [18]. Huang et al. [19] showed that PAs undergo degradation reactions at high temperatures, and Zhang et al. [20] demonstrated that the polyhydroxyl nature of PAs leads to destabilization. This is due to their degradation and interaction with other food components during frying. Under the high-temperature conditions of frying, PAs with different degrees of polymerization exhibited various levels of thermal stability. This, in turn, affects their ability to inhibit HCAs during heating. Therefore, investigating the thermal stability of PAs with different degrees of polymerization after high-temperature frying is crucial for decreasing HCA production in fried foods.

Therefore, this study used PAs with different degrees of polymerization, including highly polymerized PAs and oligomeric PAs prepared via ultrasound-assisted acid catalysis/catechin nucleophilic depolymerization. Then, the total PA and total phenolic contents, structural changes, antioxidant activity, and thermal stability of PAs with different degrees of polymerization were studied at various heating temperatures. The inhibitory rate of PAs on Norharman, Harman, and PhIP (Norharman, Harman, and PhIP are the most common HCAs) was analyzed. This study demonstrates that proanthocyanidins from Chinese quince can inhibit HCA formation in deep-frying systems and elucidates the underlying mechanism. It provides important experimental evidence on how this natural polyphenol can regulate hazardous substances in fried foods. These findings provide a theoretical basis for the development and utilization of Chinese quince proanthocyanidins as natural antioxidants and may contribute to improving the value-added utilization of Chinese quince fruit.

## 2. Materials and Methods

### 2.1. Materials

Fresh Chinese quince fruits were sourced from Zhengzhou, China. Ethanol, acetone, methanol, glacial acetic acid, petroleum ether, hydrochloric acid (37%), ammonia, and other chemicals and reagents used in this study were of analytical grade and purchased from Zhiyuan Biotechnology Co., Ltd. (Tianjin, China). Catechin, proanthocyanidin B1 (PB1), epicatechin (EC), ascorbic acid, Sephadex LH-20, AB-8 macroporous resin, sodium carbonate, Folin–Ciocalteu reagent, potassium persulfate, 2,2′-azino-bis(3-ethylbenzothiazoline-6-sulfonic acid) diammonium salt (ABTS), and 1,1-diphenyl-2-picrylhydrazyl (DPPH), PhIP (2-amino-1-methyl-6-phenylimidazo[4,5-b]pyridine, purity ≥ 98%), Harman (1-methyl-9H-pyrido[3,4-b]indole, purity ≥ 98%), and Norharman (9H-pyrido[3,4-b]indole, purity ≥ 98%) were obtained from Shanghai Yuanye Bio-Technology Co., Ltd. (Shanghai, China). Cleanert PCX chromatography columns (3 mL/60 mg) were manufactured by Agela Technologies Co., Ltd. (Tianjin, China).

### 2.2. Preparation of PAs

After washing, slicing, and deseeding, the fresh Chinese quince samples were freeze-dried at −60 °C and 17 Pa for 48 h using a freeze dryer (LGJ-12H, Beijing Sihuan Furui Scientific Instrument Co., Ltd., Beijing, China). The lyophilized Chinese quince fruits were subjected to grinding before being passed through an 80-mesh sieve to obtain a fine powder. The powder was defatted with petroleum ether for 8 h. The defatted powder (10 g) was extracted with 400 mL of mixed solvent (acetic acid/water/acetone, 0.5:29.5:70.0, *v*/*v*/*v*) under stirring for 3 h, followed by centrifugation at 4190× *g* for 10 min. The supernatant was concentrated. AB-8 macroporous (5 cm i.d. × 70 cm) resin and Sephadex LH-20 (3 cm i.d. × 70 cm) were used to purify the sample according to Lv et al. [21]. The purified sample comprised highly polymerized PAs (PPA, extraction rate: 3.73%, purity: 80.86%).

The ultrasound-assisted acid-catalyzed/catechin nucleophilic depolymerization was used to obtain oligomeric PAs (ultrasonic cleaner, GN1030, Guanbo Industrial Technology Co., Ltd., Shenzhen, China) [4]. Methanol (20 mL) was added to 50 mg of PPA, and then 50 mg of catechin was added. After adjusting the pH to 1.0 using HCl, the sample was subjected to ultrasonic-assisted treatment for 60 min at 360 W. Subsequently, the sample was concentrated, subjected to lyophilization, and collected as oligomeric PAs (OPA, purity: 44.44%).

### 2.3. Thermal Treatment of Proanthocyanidins Under Simulated Frying Conditions

The thermal stability of Chinese quince proanthocyanidins with different degrees of polymerization was evaluated according to the method of Gao et al. [22], with appropriate modifications. Oligomeric proanthocyanidins (OPA) and highly polymerized proanthocyanidins (PPA) were separately dissolved in ultrapure water at a concentration of 0.5 mg/mL. Aliquots (10 mL, containing 5 mg of PAs) were transferred into screw-capped, thick-walled, pressure-resistant reaction vials. The sealed vials were heated in an oil bath at 120, 140, 160, 180, or 200 °C for 10 min using a thermostatic magnetic stirrer (DF-101S, Lichen Bangxi Instrument Technology Co., Ltd., Shanghai, China). Unheated samples maintained at room temperature were used as controls. Immediately after heating, the vials were transferred to an ice-water bath and cooled to room temperature to terminate further thermal reactions. The residual proanthocyanidin content was subsequently determined, and the retention rate was calculated using the following equation. Three independent reaction vials were prepared for each PA type at each temperature, including the unheated control, and each replicate was heated and analyzed independently. For each PA fraction and treatment temperature, three independent samples were prepared and subjected separately to the same thermal treatment. The retention of proanthocyanidins was calculated as follows:

(1) $$\mathrm{Residual}\;\mathrm{rate}\left(\%\right)=C_{t}÷C_{0}\times 100$$

Here, C_0_ and C_t_ represent the mass concentration of PAs at time 0 and t min, respectively.

### 2.4. Compositional and Antioxidant Analyses of Thermally Treated PAs

#### 2.4.1. Determination of Total Phenol Content (TPC)

The TPC was recorded using the Folin–Ciocalteu method following the method of Wang et al. [23]. The sample (2 mL) was first diluted to 25 μg/mL, and then combined with a Folin–Ciocalteau reagent (2 mL, 1 mol/L) for 3 min. It was then reacted with sodium carbonate solution (2 mL, 7.5 g/L). The sample was placed in the dark for 90 min, followed by the measurement of absorbance at 736 nm. The result was reported in mg epicatechin (EC)/g extract.

#### 2.4.2. Determination of Total PA Content (TPAC)

According to Huo et al., the determination of TPAC in the simulated fried samples was performed through the DMAC method [24]. Water, HCl, and 91% ethanol in a ratio of 1:1:9 (*v*:*v*:*v*) were fixed in acidified ethanol. The DMAC reagent (1 mg/mL) was prepared in acidified ethanol. After mixing 3 mL of the DMAC reagent with 1 mL of the sample and incubating for 10 min, absorbance was measured at 640 nm using a UV–vis spectrophotometer (UV-6000PC, Shanghai Metash Instruments Co., Ltd., Shanghai, China). A series of proanthocyanidin B1 (PB1) standard solutions (10, 20, 40, 80, 160, 320 μg/mL) were prepared in 91% ethanol.

#### 2.4.3. DPPH Free-Radical Scavenging Assay

The DPPH scavenging rate was measured as previously described [25]. In total, 3.9 mL of 0.1 mmol/L DPPH ethanolic solution was combined with 0.1 mL of the thermally treated PA solution, followed by reaction in the dark for 30 min. Methanol and ascorbic acid (vitamin C, Vc) served as the blank and control, respectively. The absorbance of the mixture was measured at a wavelength of 517 nm.

The radical scavenging rate was computed using the following formula:

(2) $$\mathrm{DPPH}\;\mathrm{scavenging}\;\mathrm{rate}\left(\%\right)=\left[\left(A_{0}-A_{1}\right)÷A_{0}\right]\times 100$$

where A_0_ and A_1_ denote the absorbance of the blank and the sample solution, respectively.

#### 2.4.4. ABTS Free-Radical Scavenging Assay

This experiment was conducted according to the method of Wang et al. [23]. In total, 178 µL of 140 mmol/L potassium persulfate was combined with 10 mL of 7 mmol/L ABTS and was subsequently incubated for 12–16 h under dark conditions. The ABTS working reagent was diluted using phosphate-buffered saline (PBS) until an absorbance reading of 0.65–0.75 was obtained at a wavelength of 734 nm. Following the addition of 100 µL of PA solution to 3.9 mL of ABTS reagent, the reaction solution was placed in darkness for 6 min. Methanol and L-ascorbic acid (vitamin C, Vc) served as the blank and control, respectively. Absorbance detection was carried out at 734 nm. For each treatment condition, three independently heated samples were analyzed, and each sample was measured in technical triplicate.

The ABTS scavenging rate was calculated using the following formula:

(3) $$\mathrm{ABTS}\;\mathrm{scavenging}\;\mathrm{rate}\left(\%\right)=\left[\left(A_{0}-A_{1}\right)÷A_{0}\right]\times 100$$

where A_0_ and A_1_ denote the absorbance of the blank and the sample solution, respectively.

### 2.5. Structural Characterization of Thermally Treated PAs

The PPA and OPA, which were reacted at 180 °C (a common frying temperature) for 10 min, were selected and named PPA-180 and OPA-180, respectively. PPA, PPA-180, OPA, and OPA-180 were then structurally characterized.

#### 2.5.1. Determination of Mean Polymerization Degree (mDP)

The method for determining the mDP of PAs was based on the work of Wang et al. [16]. Benzyl mercaptan solution (500 µL) was taken and fixed in an HCl–methanol solution (10 mL, 0.2 mol/L) to obtain the thiolysis solution. The solution of PAs (5 mg in 1 mL chromatographic methanol) was prepared prior to heating with an equal volume of thiolysis solution at 60 °C for 2 h. The Agilent 1290II-6460 HPLC system with a diode array detector (DAD) and SB-C18 column (2.1 mm i.d. × 100 mm, 1.8 μm, Agilent Technologies, Palo Alto, CA, USA) was used for analysis. The mobile phase consisted of 0.1% formic acid aqueous solution (A) and acetonitrile (B). The gradient elution was 10–30% B (0–12 min) and was then held at 30% B (12–20 min).

The mDP of PAs was determined using the following formula:

(4) $$mDP=\frac{M}{T}+1$$

where M represents the total area of the extender units, while T corresponds to that of the terminal units.

#### 2.5.2. Fourier Transform Infrared Spectroscopy (FT-IR) Analysis

The PAs were combined with KBr in a 1:100 ratio and compressed into slices. Fourier-transform infrared (FT-IR) spectra were recorded using a Perkin–Elmer Frontier spectrometer (Perkin-Elmer, Shelton, CT, USA) over the wavenumber range of 4000–400 cm^−1^ at a resolution of 4 cm^−1^ with 32 scans.

#### 2.5.3. Gel Permeation Chromatography (GPC) Analysis

GPC (Gel permeation chromatography, Waters 1525, Waters Corporation, Milford, MA, USA) was performed following Yang et al. [26]. Separation was achieved using chromatographic-grade tetrahydrofuran (THF) as the mobile phase, with the flow rate maintained at 1 mL/min. The mixture of pyridine/acetic anhydride (5 mL, 1:1 *v*/*v*) and lyophilized PAs (20 mg) was continuously stirred in the dark for 24 h. The solution was filtered and then lyophilized. The acetylated products were redissolved in THF following drying, and the sample was obtained at a concentration of 1 mg/mL for subsequent analysis via GPC.

#### 2.5.4. Determination of Oligomer Content in PAs

The following method is referenced by Lou et al. [27]. A powder sample (20 mg) was added to 500 μL of a 70% methanol solution containing the internal standard, such that the final internal standard concentration was 0.4 mg/L. After ultrasonication for 30 min, the sample was centrifuged at 15,828× *g* at 4 °C for 5 min. In preparation for liquid chromatography–high-resolution tandem mass spectrometry (LC-MS/MS, TripleTOF 5600+, AB SCIEX™, Marlborough, MA, USA) analysis, the collected supernatant underwent filtration and was stored in a vial.

The individual PAs were eluted on a Waters ACQUITY UPLC HSS T3 C18 Column (1.8 µm, 100 mm × 2.1 mm i.d.; ANPEL, Shanghai, China). Ultrapure water with 0.05% formic acid and acetonitrile with 0.05% formic acid were mobile phases A and B, respectively. The elution gradient A/B ratio was as follows: 0 min: 90:10 (*v*:*v*), 1 min: 80:20 (*v*:*v*), 9 min: 30:70 (*v*:*v*), 12.5 min: 5:95 (*v*:*v*), 13.5 min: 5:95 (*v*:*v*), 13.6 min 90:10 (*v*:*v*), and 16 min 90:10 (*v*:*v*). The column temperature was 40 °C, the flow rate was 0.35 mL/min, and the injection volume was 2 μL.

#### 2.5.5. Thermogravimetric (TGA) Analysis

The TGA measurement was conducted at a flow rate of 60 mL/min with nitrogen purging. (Q5000 TGA, TA Instruments, New Castle, DE, USA). A total of 5 mg of PAs was used for the measurement. At a constant heating rate of 10 °C/min, the sample was measured from an initial temperature of 30 °C to a final temperature of 800 °C.

### 2.6. The Inhibitory Effect on HCA Formation

#### 2.6.1. Construction of a Simulated System for the Formation of HCAs

The simulated system for HCA formation was constructed using the method of Gao et al. [4]. The animal-protein HCA model system was as follows: For the Norharman and Harman model system, 0.1 mmol/L tryptophan, 0.1 mmol/L creatine, and 0.05 mmol/L glucose were transferred into a screw-capped tube, and 5 mg of PPA, OPA, PPA-180, and OPA-180 were added, respectively. Next, 10 mL of an 86% (*v*/*v*) diethylene glycol aqueous solution was added, and the reaction tube was transferred to a 180 °C oil bath and maintained at this temperature for 10 min. It was then placed in freezing water. For the PhIP system, tryptophan was substituted with phenylalanine under otherwise identical conditions.

The Norharman and Harman model system was designed based on the analogous animal-protein HCA model system. The plant-protein HCA model system was prepared without creatine, with all other conditions identical to those described above. The sample without PAs served as the blank control.

#### 2.6.2. Purification of HCAs

The method of purification referenced is from Zhang et al. [28]. Cleanert PCX columns (Bonna-Agela Technologies, Tianjin, China) were activated with 3 mL of methanol and 3 mL of water, respectively. Following the addition of 0.5 mL HCA solution, the column was washed sequentially with 3 mL of 0.1 mol/L HCl and 3 mL of methanol. Elution was subsequently carried out with 3 mL of a 95:5 (*v*/*v*) methanol–ammonia solution. The eluents were evaporated to dryness with N2. Afterward, methanol (1 mL) was added to the samples, followed by filtration through a 0.22 μm polypropylene spin filter.

#### 2.6.3. Determination of HCA Content

The content of HCAs was detected following the procedure by Wang et al. [16]. The chromatographic conditions were as follows: the column: Waters ACQUITY UPLC BEH C18 (50 mm × 2.1 mm, 1.7 μm); mobile phase A: ammonium formate (10 mmol/L; pH = 6.80); mobile phase B: acetonitrile; elution gradient: mobile phase B ratio: 0–0.2 min, 10%; 0.2–1.0 min, 30%; 1.0–3.0 min, 60%; 3.0–3.5 min, 90%, balanced to initial state; flow rate: 0.3 mL/min.

The MS conditions were as follows: the ion source was electrospray ionization (ESI) in positive ion mode; the capillary voltage was 0.5 kV; and the ion source temperature was 110 °C. The desolvation temperature parameters were as follows: 650 °C; desolvation gas flow (N_2_) of 1000 L/h; tapered gas flow (N_2_) of 50 L/h; and collision gas (argon). HCAs were calculated according to the following formula:

(5) $$IR\left(\%\right)=\left[\left(A_{C}-A_{t}\right)÷A_{C}\right]\times 100\%$$

The values for Ac and At represent the levels in the blank control group and the group supplemented with PAs, respectively.

### 2.7. Statistical Analysis

All measurements were performed in triplicate and the results were expressed as means ± standard deviations (SDs). One-way ANOVA was conducted using SPSS 25.0 (SPSS Inc., Chicago, IL, USA). Significance testing was conducted using Duncan’s multiple range test. When *p* < 0.05, the difference was statistically significant.

## 3. Results

### 3.1. Analysis of Mean Degree of Polymerization

PAs with different degrees of polymerization were prepared to simulate frying. Muñoz-Labrador et al. used ultrasound to depolymerize grape PAs [29]. Liu et al. used (+)-catechin, (-)-epigallocatechin, and (-)-epigallocatechin gallate as chain-breakers to depolymerize cranberry PAs [30]. Ultrasound and acid-catalyzed degradation were commonly used methods. The nucleophilic reagent not only degraded polymers without introducing new impurities, but also allowed selective synthesis of target products [31]. Consequently, oligomeric PAs were prepared via ultrasound-assisted acid-catalyzed/catechin nucleophilic depolymerization. Thiolysis-based efficient depolymerization enabled detection of the structure and mDPs of PAs [26]. In the presence of nucleophilic reagents, the primary structure of PAs can be characterized by analyzing the extension and terminal units released upon depolymerization [27].

In Figure 1A–D, Peak 1 was identified as catechin (289 Da, [M−H]−) and Peak 2 as epicatechin (289 Da, [M−H−]), both of which served as terminal units. Peaks 3, 4, 5, 6, 7, and 8 were identified as extension units, all of which were (epi)catechin benzyl thioethers (411 Da, [M−H]−). As shown in Table 1, the degrees of polymerization for PPA, PPA-180, OPA, and OPA-180 were 41.86, 27.45, 4.03, and 1.88, respectively. The results demonstrated that PPA was a highly polymerized PA and OPA was an oligomer. Previous studies have confirmed that the C-C interflavan bonds present in PAs can be cleaved under acidic conditions [32]. Under acidic conditions, highly polymerized PAs were effectively depolymerized into oligomeric PAs, using catechin as a chain breaker [30].

The data showed that the mDP of PAs decreased significantly after simulated frying at 180 °C, with a more pronounced reduction observed in PPA. This may be because the interflavan bonds in PAs depolymerize under high temperatures, leading to the formation of new oligomeric PAs [33]. In PPA, a greater proportion of polymers depolymerize at high temperatures, resulting in a more significant change in mDP.

### 3.2. Analysis of TPAC, TPC, and Residual Rate

The TPAC (Figure 2A) and TPC (Figure 2B) of OPA and PPA were reduced after heat treatment at different temperatures. This suggested that PAs degraded during thermal processing. This phenomenon could be attributed to the PA skeleton’s tendency to undergo opening, hydration, and oxidative decomposition under high-temperature conditions [34], leading to the destruction of PA and phenolic compound hydroxyl groups [35]. In addition, the structural changes in PAs significantly influenced the resulting molar absorption coefficients, leading to distinct differential absorbance responses [36]. Consequently, TPAC and TPC measured by the colorimetric method decreased after structural disruption.

The residual rate of PAs after simulated frying is calculated based on the TPAC. Its changing nature reflects their thermal stability. Figure 2C demonstrates that the residual rates of OPA and PPA decreased with increasing temperature. For frying temperatures below 140 °C, the residual rate of PPA was higher than that of OPA. However, when the frying temperature exceeded 140 °C, OPA showed a significantly higher residual rate than PPA, and the decrease in OPA and PPA was most notable at 140–160 °C. Notably, OPA maintained a residual rate exceeding 70% at a 180 °C frying temperature. The results indicated that OPA retained a higher content of PAs at high temperatures. This phenomenon resulted from the highly polymerized PAs readily coalescing and precipitating under high-temperature conditions [22]. Thus, it was demonstrated that oligomeric PAs can be more effectively utilized as a natural antioxidant in practical frying thermal systems than highly polymerized PAs.

### 3.3. Antioxidant Activity Analysis

To evaluate the antioxidant activity of PAs, we used DPPH and ABTS radical scavenging assays. The DPPH (Figure 3A) and ABTS (Figure 3B) radical scavenging rates of both OPA and PPA declined with increasing temperature, and their free-radical scavenging capacities were significantly different at various temperatures. OPA’s antioxidant capacity was generally much better than that of PPA, because the mDP of PAs was inversely correlated with antiradical activity [37]. The above results showed that heat treatment reduced the antioxidant capacity of PAs. This was due to the significant reduction in TPAC and TPC detected in the samples after simulated frying [23,38]. The findings are consistent with previous studies. Wu et al. demonstrated that thermal treatment significantly reduced both DPPH and ABTS scavenging activities of anthocyanin solutions, with more pronounced effects at higher temperatures [39].

Although the antioxidant capacity of both OPA and PPA decreased after simulated frying, OPA still had strong antioxidant activity at 180 °C (a common frying temperature). The free-radical scavenging rates of DPPH and ABTS reached 50.40% and 26.30%, respectively, which were higher than PPA’s scavenging rates of 29.68% and 9.10% at the same temperature. Both were much higher than the scavenging rates of the control Vc. These results show that OPA has excellent antioxidant capacity.

### 3.4. Structural Characterization of PAs

#### 3.4.1. FT-IR Spectroscopy Analysis

In Figure 4A,B, the FT-IR spectra of PPA and OPA before and after the simulated frying reaction are presented. The prominent absorption band close to 3385 cm^−1^ was attributed to the stretching vibration of the hydroxyl group in PAs [40]. For OPA and OPA-180, the absorption peak in the vicinity of 1690 cm^−1^ was assigned to the C=O stretching vibration, indicating the presence of galloyl groups. Characteristic peaks of aromatic benzene rings appeared at 1430–1620 cm^−1^. Therefore, the peaks near 1442, 1522, and 1610 cm^−1^ correspond to the vibrational peaks of the benzene ring skeleton [28]. The absorption peaks from 1060 to 1280 cm^−1^ were regarded as characteristic peaks corresponding to C-O-C stretching vibrations in PA carbon rings [27]. The peak at approximately 822 cm^−1^ was attributed to the presence of three H atoms on the benzene ring, while the peak at 781 cm^−1^ was due to the C-H planar structure of the carbon ring in B-type procyanidins. This indicates the existence of B-type procyanidin structures in both high-molecular-weight and low-molecular-weight polymers of PAs [41]. Comparative analysis of PPA, PPA-180, OPA, and OPA-180 in Figure 4A and Figure 4B revealed that their characteristic peaks and absorption intensities remained highly similar, demonstrating that the basic structures of PPA-180 and OPA-180 remained similar to PPA and OPA, respectively. This indicates that the core structures of PPA and OPA remained largely unchanged upon exposure to high temperatures in the simulated frying system, and their functional groups were not altered.

#### 3.4.2. GPC Analysis

The properties of PAs with different degrees of polymerization (e.g., thermal stability and antioxidant activity) vary significantly, primarily due to differences in molecular weight [40]. As shown in Figure 4C,D, the molecular weights of PAs with different degrees of polymerization before and after the simulated frying reaction were investigated using GPC. The weight-average (Mw) and number-average (Mn) molecular weights, along with the polydispersity index (PDI) of the PAs, are summarized in Table 1.

Based on Table 1, the Mw and PDI follow a descending order: PPA > PPA-180 > OPA > OPA-180. The decrease in both Mw and PDI of PPA and OPA indicated that depolymerization was the predominant reaction pathway [42]. This was consistent with the mDP results. Highly polymerized PAs were degraded into oligomeric PAs at high temperatures, resulting in PAs containing more small molecules. Therefore, after the high-temperature reaction at 180 °C, the intermolecular chemical bonds of PAs broke down [27]. The molecular weight distribution of PAs was characterized using PDI values; a lower PDI value indicated a narrower molecular weight distribution [26]. Combining Figure 4C and Figure 4D shows that the molecular weight distributions of PPA and PPA-180 were greater than those of OPA and OPA-180, respectively. The PDI of OPA-180 was 1.60. Compared with OPA, PPA, and PPA-180, OPA-180 exhibited the lowest PDI value, indicating a smaller molecular weight distribution. The relatively low PDI further demonstrated its excellent physicochemical properties [43] and confirmed that OPA-180 exhibited higher uniformity and stability.

#### 3.4.3. LC-MS/MS Analysis

Table 2 shows the oligomeric composition of PPA, PPA-180, OPA, and OPA-180. Most detected components, including catechin, epicatechin, gallocatechin, epigallocatechin, (epi)gallocatechin gallate, (epi)catechin gallate, and procyanidin B2, align with findings from studies on Chinese quince fruits [14]. After the simulated frying reaction at 180 °C, the oligomeric components of PPA and OPA showed an upward trend. Among them, the oligomeric content of PPA increased from 1519.56 μg/g to 3061.02 μg/g, and that of OPA increased from 8579.86 μg/g to 16,783.16 μg/g. This showed that the cleavage of C-C bonds in PAs at high temperatures resulted in the formation of more oligomeric PAs [32]. In addition, OPA and OPA-180 demonstrated greater oligomeric content than PPA and PPA-180, respectively (Table 2). This phenomenon was attributed to the ultrasound-assisted acid depolymerization treatment applied to OPA, which resulted in a higher oligomeric PA content [31].

#### 3.4.4. Thermal Stability Analysis

To further understand the thermal stability of PPA and OPA, TG and DTG measurements were conducted from 30 to 800 °C. Due to the differences in the physical properties and chemical composition of OPA and PPA structural components, variations in thermal stability were observed [44]. This is one of the main reasons for the wider range of PA thermal weight loss.

The maximum heat loss rate (DTGmax) curve reflects the thermal stability of PAs. In the TG curve (Figure 5), three distinct stages emerge: the first stage (50–150 °C) is attributed to the evaporation of water and loss of other small molecular substances; the second stage (200–300 °C) exhibits significant mass loss due to the thermal degradation of PA components at the temperature interval of 500–700 °C; and the third stage is characterized by a slow mass loss, attributed to the carbonization of residual weight [22]. The DTG curves for all samples show mass loss during these three stages. The higher temperature for 50% weight loss in PAs corresponds to superior thermal stability [44]. At 50% weight loss, the decomposition temperatures of PPA, PPA-180, OPA, and OPA-180 were 547.06 °C, 541.93 °C, 539.46 °C, and 492.48 °C, respectively. At 800 °C, PPA, PPA-180, OPA, and OPA-180 retained high final residual weights of 42.10%, 41.24%, 41.34%, and 38.47%, respectively. The PPA and OPA exhibited superior thermal stability compared to PPA-180 and OPA-180, respectively. Therefore, the results demonstrate that thermal stability was higher in PAs that did not undergo simulated frying.

### 3.5. HCA Content Analysis

In the model frying system, the ability of PPA, OPA, PPA-180, and OPA-180 to inhibit the generation of Norharman, Harman, and PhIP was assessed. As shown in Figure 6A,B, in the animal-protein HCA model system, PPA, OPA, PPA-180, and OPA-180 exhibited optimal inhibitory effects on Norharman. By contrast, in the plant-protein HCA model system, they showed the highest efficacy against Harman. In the animal- and plant-protein HCA model systems, OPA inhibited different types of HCAs more than PPA. This may be attributed to the higher TPAC and TPC, as well as the higher free-radical scavenging capacity of OPA. Due to their high antioxidant capacity, PAs were effective in removing intermediates (carboxylates, pyridines, and pyrazines) in the Maillard reaction. Thus, they inhibited the production of HCAs more effectively [10]. PAs with low mDP contained more active sites than those with high mDP at the same mass concentration [33]. Active sites can better scavenge the free radicals generated during the Maillard reaction and reduce the number of HCAs generated more effectively. Wang et al. showed that PAs with low degrees of polymerization exhibited a stronger inhibitory effect against HCAs. In the animal- and plant-protein HCA model system, the inhibitory effects of PPA-180 and OPA-180 on HCAs were stronger than those of PPA and OPA, respectively [16]. This was attributed to the cleavage of the interflavanic bonds in PAs under high temperatures, which led to the formation of new oligomeric PAs [33]. In the animal-protein HCA model system, OPA-180 most effectively inhibited Norharman (85.99%), Harman (80.05%), and PhIP (82.58%), whereas in the plant-protein HCA model system, OPA-180 best inhibited Norharman (74.66%) and Harman (79.70%). This was related to the lowest mDP observed in OPA-180.

### 3.6. Correlation Analysis

Correlation analysis was employed to evaluate the antioxidant capacity (DPPH, ABTS), thermal stability (retention rate), and inhibitory effect of HCAs (Noharman, Harman, PhIP) on PAs following heat treatment at different mDPs. As shown in Figure 7, TPAC, TPC, and oligomeric PA contents positively correlated with antioxidant capacity. Zhang et al. indicated that antioxidant capacity increased with rising TPC and TPAC [45]. This may be because PAs with higher phenolic hydroxyl content possessed greater free-radical scavenging capacity [46]. Bordiga et al. found that the antioxidant activity of PAs was inversely proportional to mDP, consistent with our findings [38]. The mDP, Mw, and thermal stability (residual weight) showed a negative correlation with HCA inhibition. The content of oligomeric PAs exhibited a positive correlation with HCA inhibition. The thermal stability analysis, expressed as residual weight, is consistent with the TGA results. This correlation likely results from the thermal degradation of PAs under high temperatures, which generates more oligomeric PAs and consequently reduces their mDP and Mw. Oligomeric PAs exhibited superior free-radical scavenging capacity, thereby enhancing the inhibition rate of HCAs. Moreover, at equivalent mass, PAs with lower mDP possessed more active sites compared with those with higher mDP [33]. These PAs can inhibit HCA formation by creating adducts with HCA intermediates [16].

## 4. Conclusions

In this study, PPA and OPA were subjected to thermal treatment at different temperatures under frying-relevant conditions. The TPAC, TPC, and residual rates of both PPA and OPA decreased with increasing temperature, with OPA consistently showing higher values than PPA. At 180 °C, the residual rate of OPA remained above 70%. The antioxidant activities of both fractions also decreased as the temperature increased, although OPA exhibited stronger antioxidant activity than PPA. Structural analysis showed that high-temperature treatment did not markedly alter the core structures of PPA and OPA, whereas the total oligomer contents of PPA-180 and OPA-180 increased compared with their corresponding untreated fractions. OPA and OPA-180 exhibited stronger inhibition of HCA formation than PPA and PPA-180, respectively. In addition, both thermally treated fractions showed greater inhibitory activity than their untreated counterparts. Among the tested samples, OPA-180 exhibited the strongest inhibitory activity, with inhibition rates of 85.99%, 80.05%, and 82.58% against Norharman, Harman, and PhIP, respectively, in the animal-protein HCA model system, and 74.66% and 79.70% against Norharman and Harman, respectively, in the plant-protein HCA model system. These results suggest that the degree of polymerization and thermal treatment influence the properties and HCA-inhibitory activity of Chinese quince proanthocyanidins. OPA, particularly thermally treated OPA, therefore shows potential as a natural functional ingredient for reducing HCA formation in thermally processed protein-rich foods. Further studies in actual food systems are needed to confirm these effects and evaluate sensory quality, safety, and appropriate application levels.

## Figures and Tables

**Figure 1:**
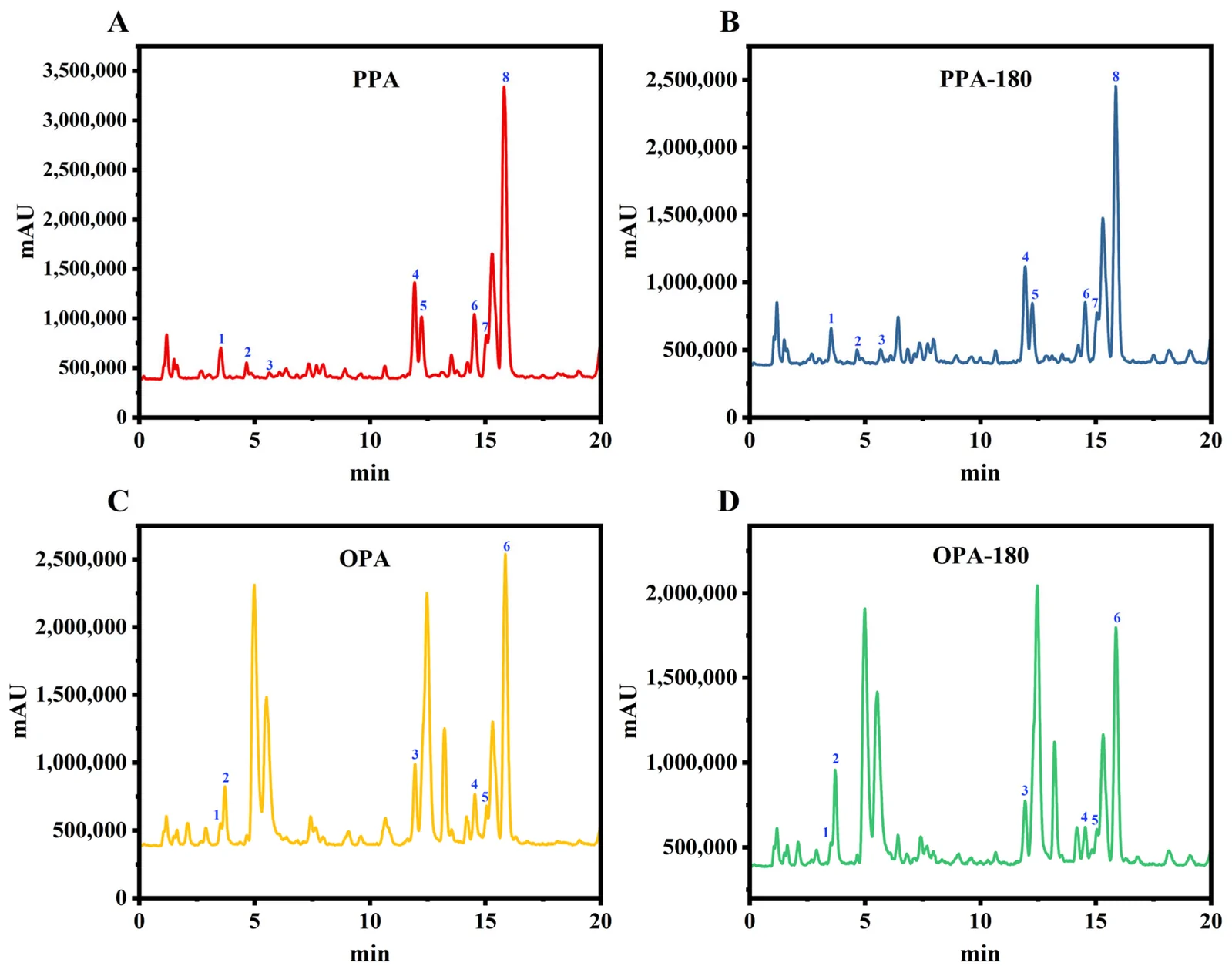
RP-HPLC chromatograms of proanthocyanidin thiolysis products. (**A**) PPA, highly polymerized PAs; (**B**) PPA-180, PPA heated at 180 °C; (**C**) OPA, oligomeric PAs; (**D**) OPA-180, OPA heated at 180 °C.

**Figure 2:**
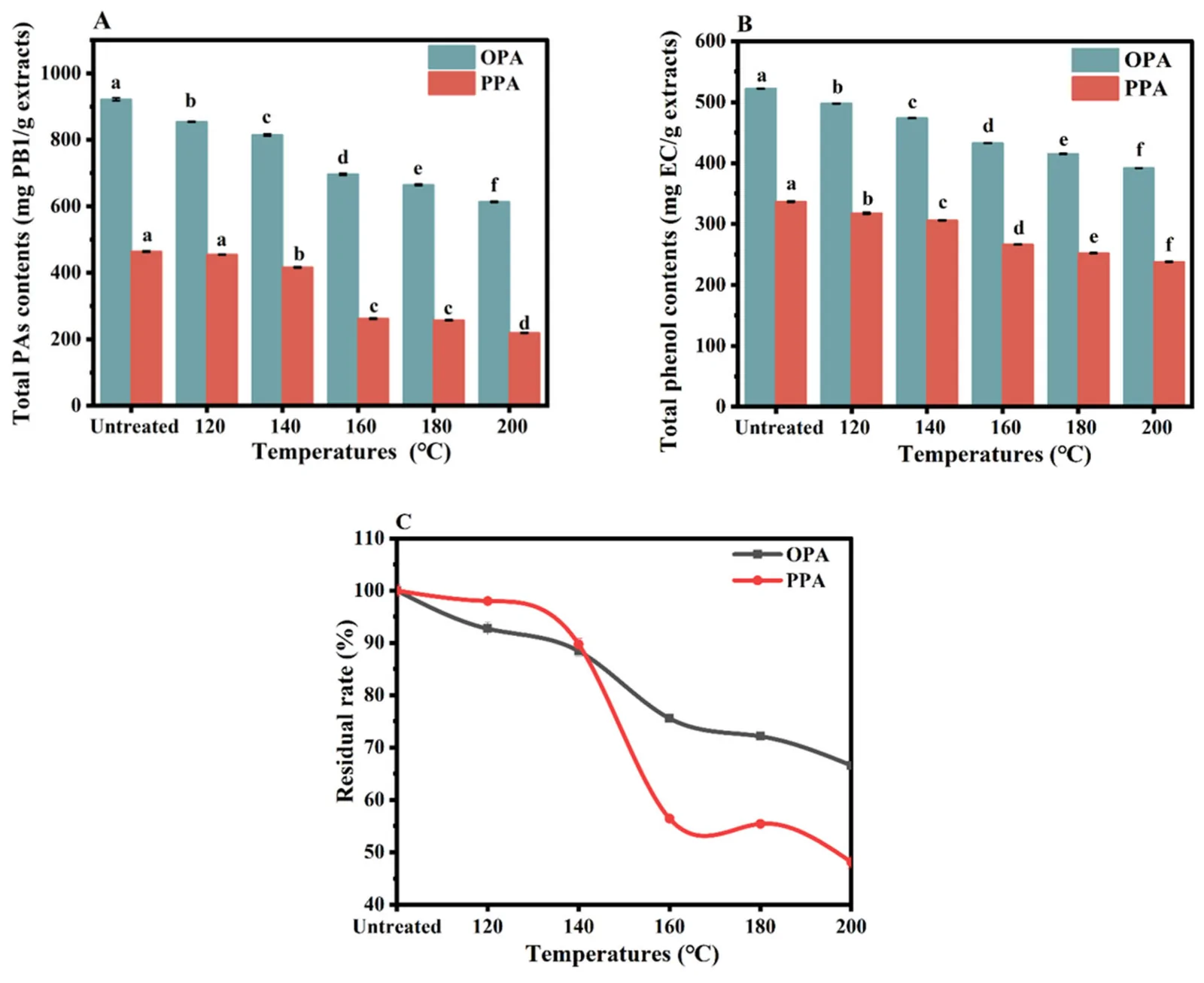
Total PAs (**A**), total phenol (**B**) contents, and residual rate (**C**) with different polymerization degrees before and after simulated frying at different temperatures. Means of the same index with different lowercase letters differ significantly (*p* < 0.05). The significance marker compares uniform treatments of different samples.

**Figure 3:**
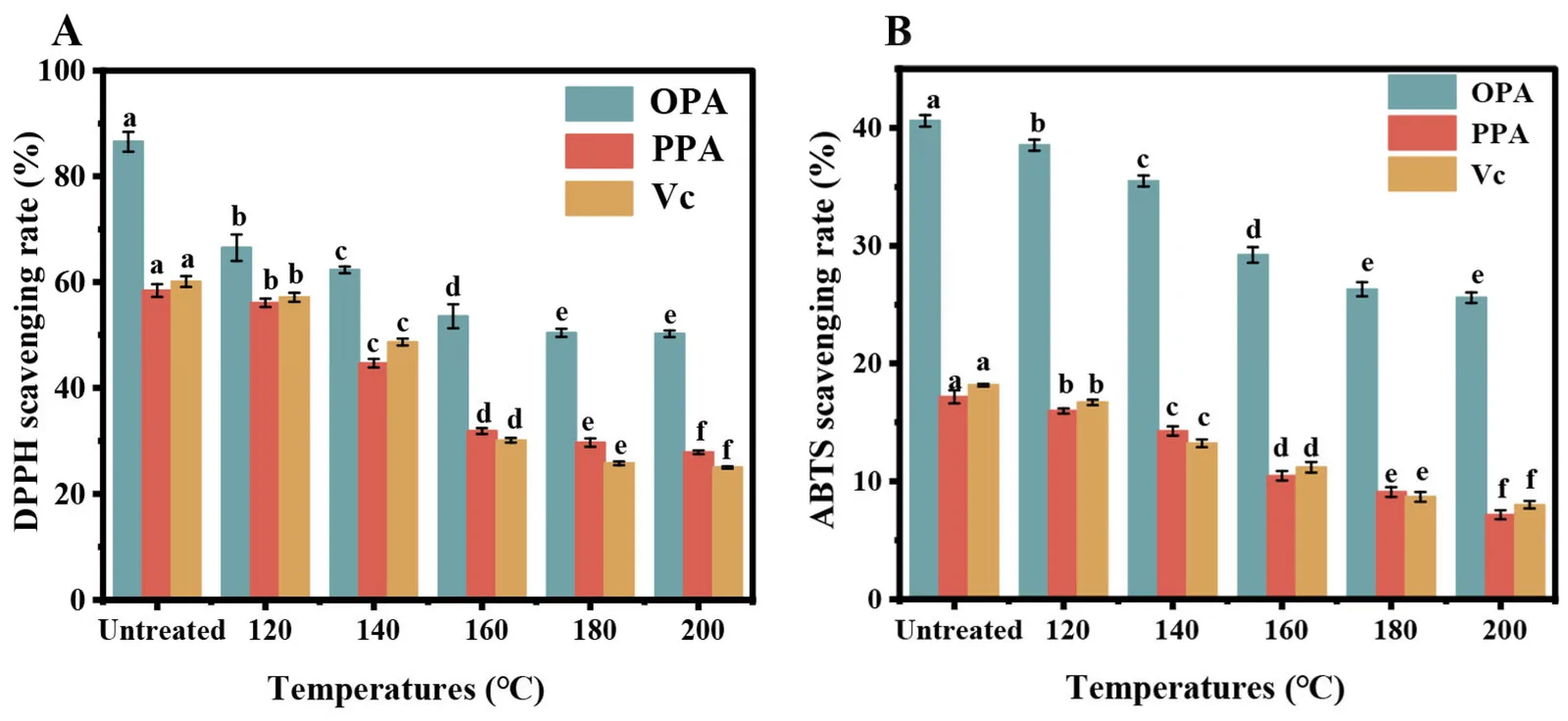
DPPH free-radical (**A**) and ABTS (**B**) free-radical scavenging abilities of PAs before and after simulated frying reactions at different temperatures. Means of the same index with different lowercase letters differ significantly (*p* < 0.05). VC: vitamin C (ascorbic acid).

**Figure 4:**
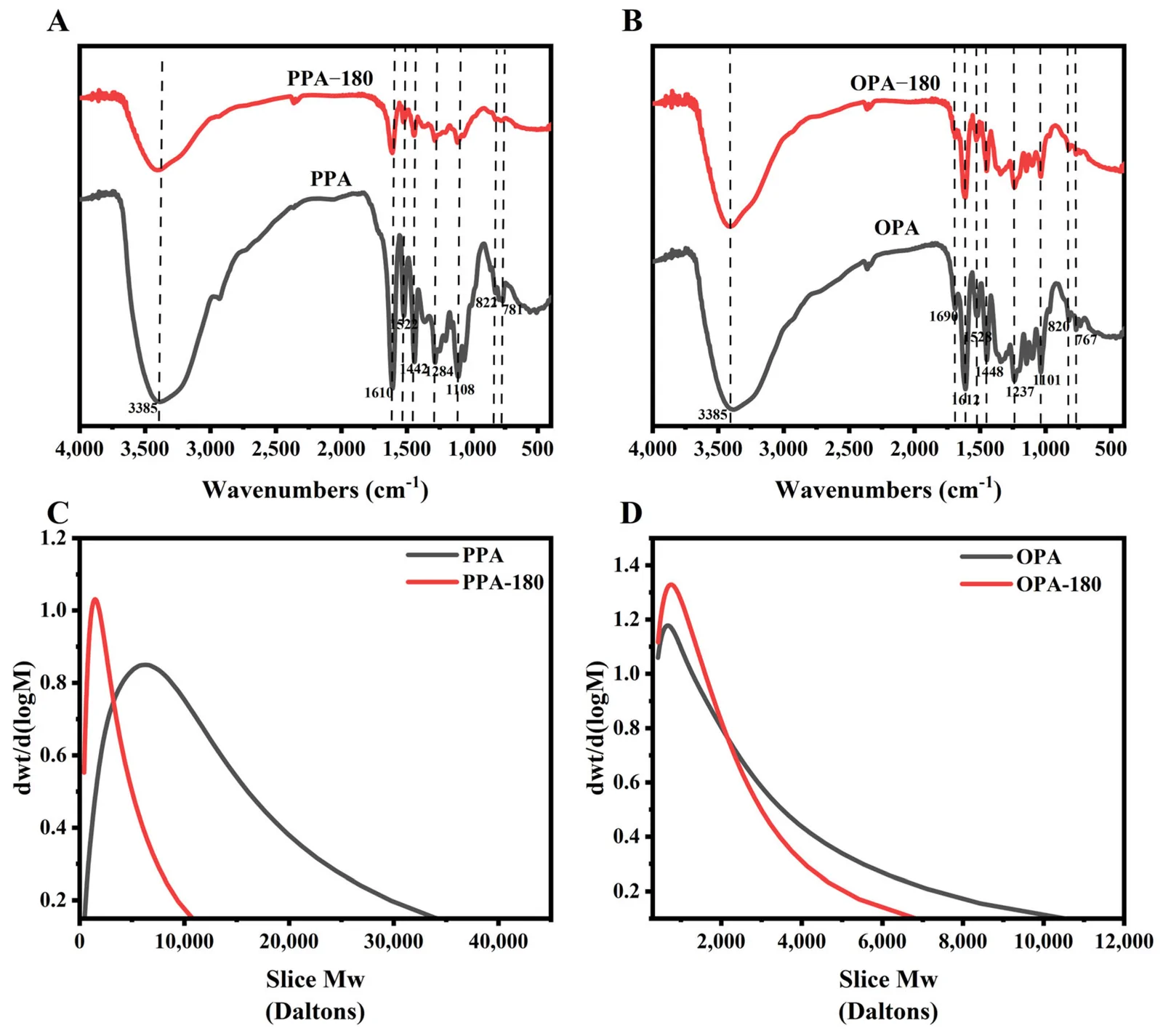
Fourier transform infrared spectroscopy of PPA (**A**) and OPA (**B**) before and after the simulated frying reaction; molecular weight distribution curves of PPA (**C**) and OPA (**D**) before and after the simulated frying reaction.

**Figure 5:**
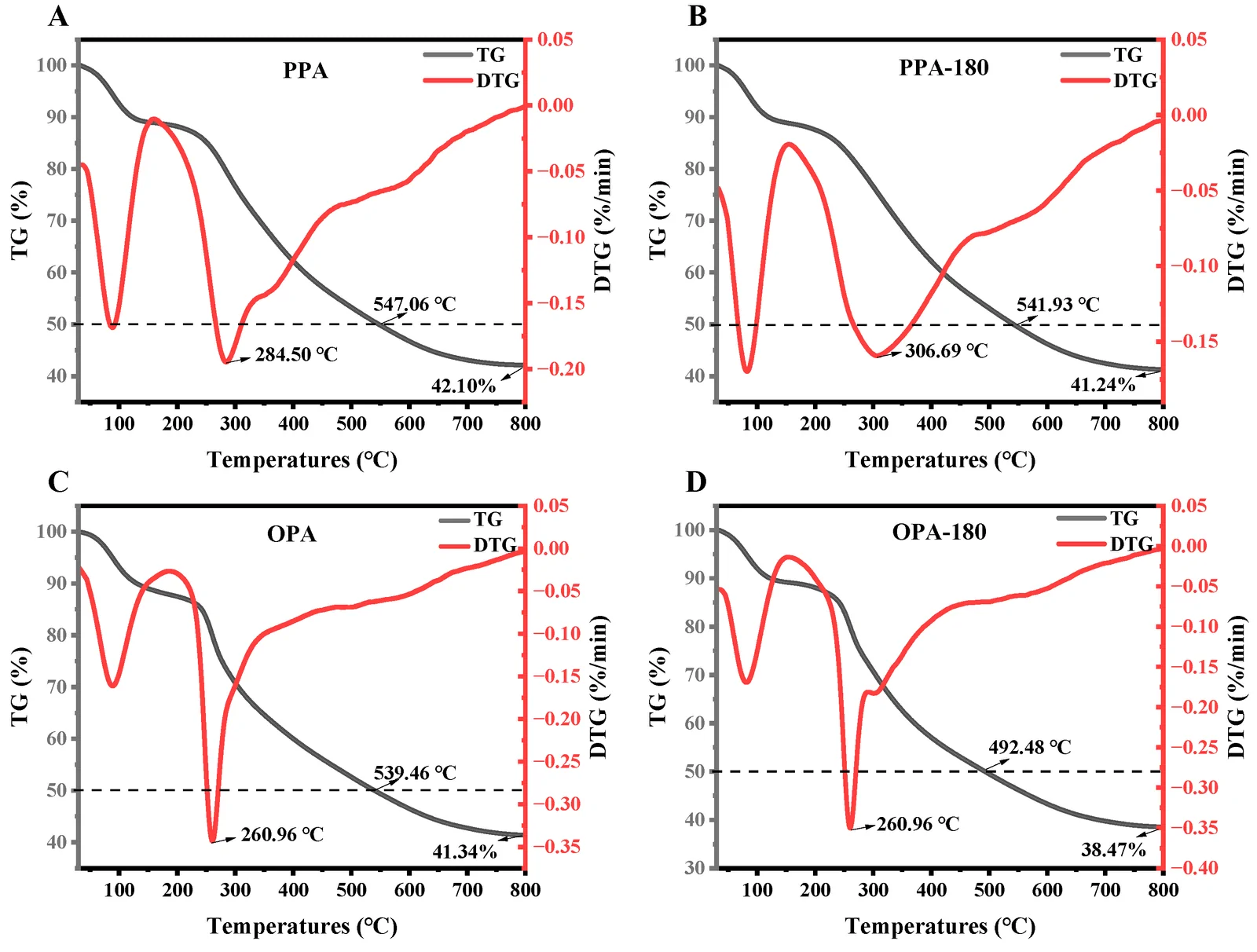
Thermogravimetric (TG) and derivative thermogravimetric (DTG) curves before (**A**) and after (**B**) the simulated frying reactions of PPA and (**C**,**D**) OPA.

**Figure 6:**
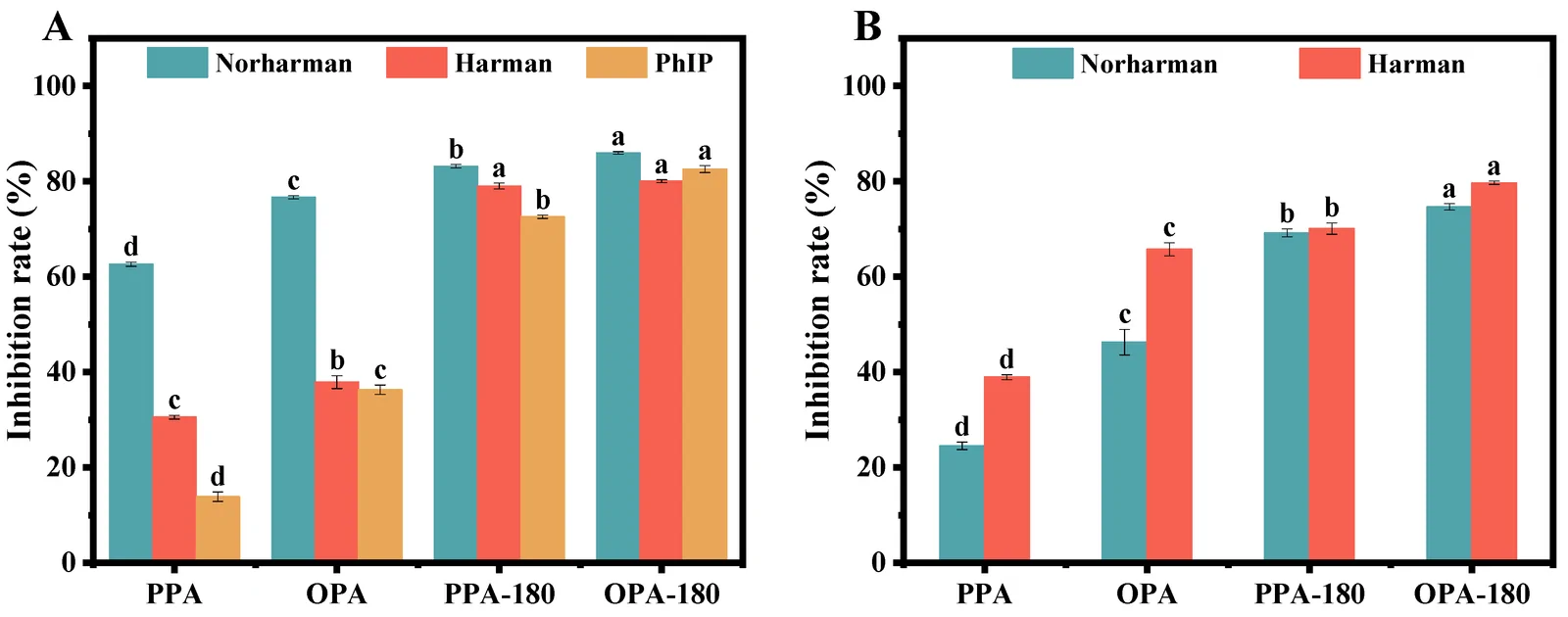
Effect of adding PPA, OPA, PPA-180, and OPA-180 on HCA content in animal-protein (**A**) and plant-protein (**B**) HCA model frying systems. Means of the same index with different lowercase letters differ significantly (*p* < 0.05).

**Figure 7:**
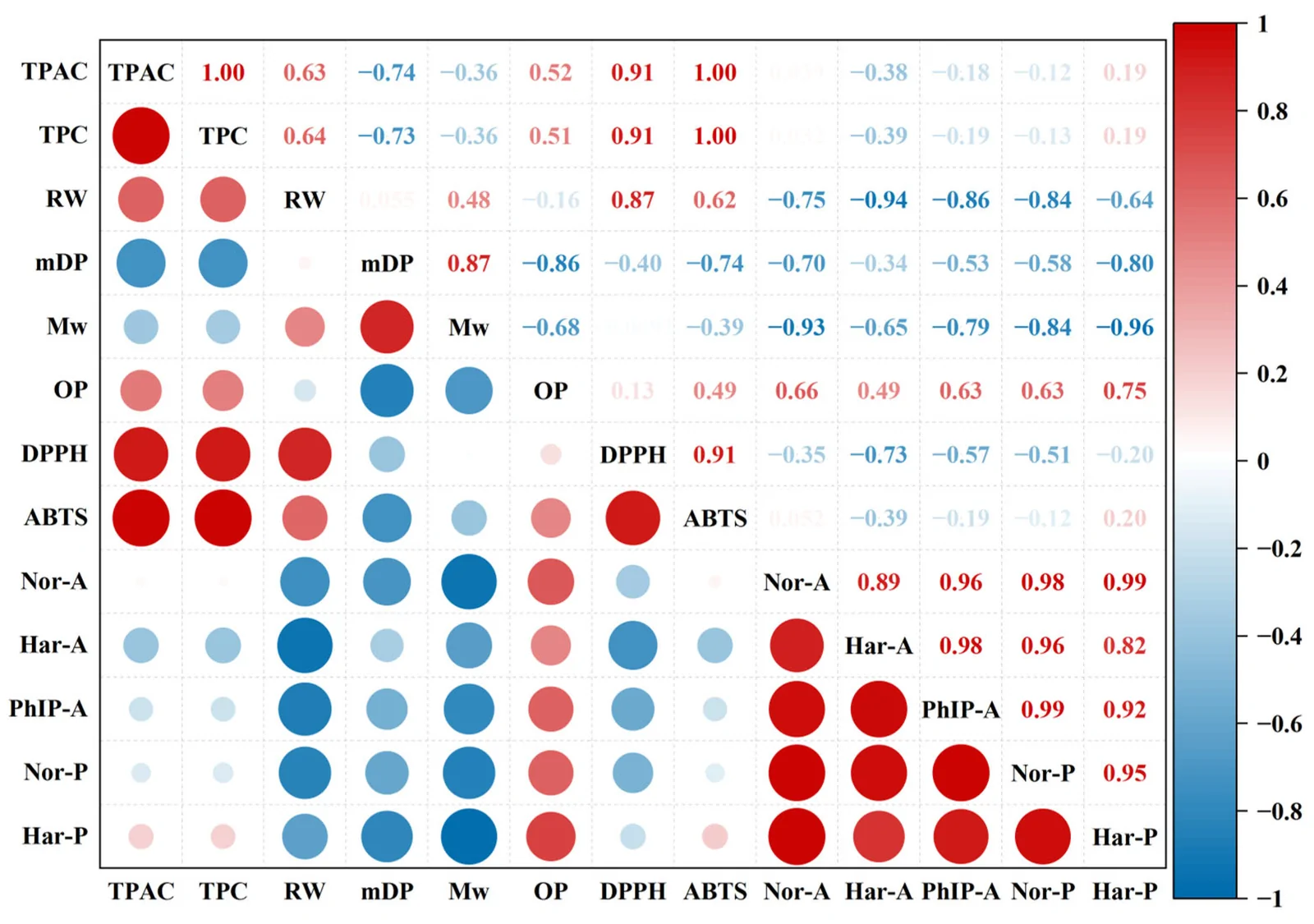
Correlation analysis plots for PAs and HCAs. TPAC: total PA content; TPC: total phenol content; RW: residual weight; mDP: mean degree of polymerization; Mw: average molecular weight; OP: oligomeric PAs; Nor-A, Har-A, and PhIP-A: inhibition rates of Norharman, Harman, and PhIP in the animal-protein HCA model system; Nor-P and Har-P: inhibition rates of Norharman and Harman in the plant-protein HCA model system. The size of the circles represents the absolute magnitude of the correlation coefficient, with larger circles indicating stronger correlations.

**Table 1:** Composition units, mean polymerization degree, and molecular weight of PPA, PPA-180, OPA and OPA-180.

	PPA	PPA-180	OPA	OPA-180
mDP	41.86 ± 3.70 ^a^	27.45 ± 0.78 ^b^	4.03 ± 0.08 ^c^	1.88 ± 0.01 ^c^
Terminal unit				
Catechin (mass%)	1.80 ± 0.15 ^b^	2.64 ± 0.02 ^a^	1.05 ± 0.01 ^c^	1.14 ± 0.02 ^c^
Epicatechin (mass%)	0.60 ± 0.06 ^c^	1.03 ± 0.01 ^c^	23.74 ± 0.09 ^b^	52.03 ± 0.51 ^a^
Extension unit				
Catechin/epicatechin (mass%)	97.60 ± 0.21 ^a^	96.33 ± 0.10 ^b^	75.21 ± 0.50 ^c^	46.83 ± 0.17 ^d^
*M_n_* (Daltons)	2730 ± 108.50 ^a^	1306 ± 103.00 ^b^	1032 ± 47.00 ^c^	955 ± 62.50 ^c^
*M_w_* (Daltons)	7597 ± 393.50 ^a^	2607 ± 101.50 ^b^	1999 ± 133.50 ^c^	1523 ± 93.00 ^c^
PDI (*M_w_*/*M_n_*)	2.78 ± 0.03 ^a^	2.00 ± 0.08 ^b^	1.94 ± 0.04 ^b^	1.60 ± 0.01 ^c^

Values with different letters in the same row differ significantly (*p* < 0.05). The data are presented as the average of three measurements.

**Table 2:** The main compounds of PAs fraction.

Compounds	Contents (μg/g Extract)			
	PPA	PPA-180	OPA	OPA-180
Catechin	ND ^d^	216.18 ± 12.46 ^a^	125.70 ± 7.16 ^c^	174.49 ± 8.52 ^b^
Epicatechin	47.24 ± 2.51 ^a^	ND ^c^	34.28 ± 1.11 ^b^	ND ^c^
gallocatechin	ND ^b^	ND ^b^	ND ^b^	610.86 ± 19.21 ^a^
Epigallocatechin	3.34 ± 0.15 ^c^	1.92 ± 0.07 ^c^	486.42 ± 24.34 ^a^	408.23 ± 15.11 ^b^
(Epi) catechin gallate	ND ^c^	ND ^c^	119.31 ± 7.67 ^a^	62.67 ± 3.26 ^b^
(Epi) gallocatechin gallate	55.43 ± 1.77 ^c^	74.83 ± 4.13 ^c^	7185.54 ± 255.41 ^b^	15,019.51 ± 283.37 ^a^
Procyanidin B2	1413.55 ± 51.20 ^b^	2768.08 ± 92.30 ^a^	628.61 ± 31.30 ^c^	507.41 ± 26.11 ^d^
Oligomeric procyanidins	1519.56 ± 51.78 ^d^	3061.02 ± 108.95 ^c^	8579.86 ± 326.93 ^b^	16,783.16 ± 325.29 ^a^

The data are presented as the average of three measurements. ND indicates not detected. Values with different letters in the same row differ significantly (*p* < 0.05).

## Data Availability

The original contributions presented in the study are included in the article. Further inquiries can be directed to the corresponding authors.

## Abbreviations

The following abbreviations are used in this manuscript:

Abbreviation	Full name
PAs	Proanthocyanidins
PB1	Proanthocyanidin B1
ABTS	2,2′-azino-bis (3-ethylbenzothiazoline-6-sulfonic acid) diammonium salt
DPPH	1,1-diphenyl-2-picrylhydrazyl
PPA	Polymerized PAs
OPA	Oligomeric PAs
TPC	Total phenol content
EC	Epicatechin
Vc	Vitamin C (Ascorbic acid)
GPC	Gel permeation chromatography
THF	Tetrahydrofuran
TGA	Thermogravimetric analysis
ESI	Electrospray ionization
